# The Use of Percutaneous Stent-Kyphoplasty (SpineJack®) in Osteoporotic and Non-Osteoporotic Vertebral Fractures: A Retrospective Analysis of 310 Implants From a Level-1 Trauma Center in Switzerland

**DOI:** 10.1177/21925682251347225

**Published:** 2025-05-29

**Authors:** Magdalena Karner, Georg Osterhoff, Kai Sprengel, Hans-Christoph Pape, Julian Scherer

**Affiliations:** 1 Department of Traumatology, 27243 University Hospital of Zurich, Zurich, Switzerland; 2 Department of Orthopaedics, Trauma and Plastic Surgery, University Hospital Leipzig, Leipzig, Germany; 3 Praxis medOT, Hirslanden Clinic St. Anna, Lucerne, Switzerland; 4 Orthopaedic Research Unit, University of Cape Town, Cape Town, South Africa

**Keywords:** kyphoplasty, SpineJack®, vertebral compression fracture, spine

## Abstract

**Study Design:**

Retrospective cohort study.

**Objectives:**

The SpineJack®-system represents a new generation of kyphoplasty for the treatment of traumatic and osteoporotic fractures. The aim of this study was to analyze the usage, safety and efficacy of the SpineJack®-system, in both osteoporotic and non-osteoporotic fractures.

**Methods:**

310 patients with vertebral fractures treated with the SpineJack®-system between November 2014 and December 2022 were analyzed. Demographics, intraoperative parameters and outcomes were assessed. A subgroup analysis was performed for traumatic vertebral fractures (tVCFs) and osteoporotic vertebral fractures (oVCFs).

**Results:**

SpineJack®-kyphoplasty was performed for 157 (47.4%) oVCFs and 153 (46.2%) tVCFs. Stand-alone SpineJack®-kyphoplasty was performed in 128 patients, while 182 patients underwent combined procedures. The mean pain reduction from admission to discharge was 3.8 (range 3-10, SD 2.7). Opioids were administered for an average of 4.6 days (range 0-72 days, SD 7.2 days). The overall mean hospital length of stay was 6.1 days, significantly shorter for patients undergoing stand-alone SpineJack® procedures at 4.8 days (SD 6.9 days). The most common complication observed was cement extravasation in 29 patients (8.8%), followed by neurological symptoms in 8 patients (2.4%) and surgical site infections in 4 patients (1.2%). Notably, no surgical site infections were recorded in the stand-alone SpineJack®-group.

**Conclusion:**

Percutaneous stent-kyphoplasty (SpineJack®-system) appears to be a safe and effective therapeutic option, whether used as a stand-alone procedure or in combination with other interventions, for osteoporotic and non-osteoporotic vertebral fractures.

## Introduction

Vertebral compression fractures reflect a common orthopaedic problem and are either caused by high-energy trauma in younger patients (tVCF) or low-energy trauma in (elderly) patients with osteoporosis (oVCF).^1-3^

The clinical symptoms may include back pain, increase of pain intensity while standing or walking and functional impairment. Furthermore, studies have demonstrated that VCFs have a negative impact on mental health, mortality and quality of life (QoL).^1,4,5^

X-rays, CT-scan and magnetic resonance imaging (MRI) are the primary imaging modalities used for diagnosis and evaluation of extent (instability, discoligamentous injuries) in patients with trauma to the vertebral column.^6^

The AOSpine thoracolumbar fracture classification system (tVCF) and the AOSpine-DGOU Osteoporotic Fracture classification system (oVCF) are well validated classification systems which also aim to aid in the treatment decision.^7-10^ The cornerstone in the treatment of vertebral fractures is appropriate analgesia and fast mobilization. Depending on the severity of vertebral fractures, treatment options include conservative management including bracing, vertebroplasty, balloon-kyphoplasty, or more invasive surgical procedures like dorsal instrumentation or fusions. Conventional vertebroplasty and balloon-kyphoplasty have shown good results in the treatment of Type A fractures.^11-16^

Both vertebroplasty and kyphoplasty are considered relatively safe minimally invasive procedures with low peri- and postoperative complications.^13,17-19^

The SpineJack®-system represents a new generation of kyphoplasty (percutaneous stent-kyphoplasty) for the treatment of osteoporotic and non-osteoporotic fractures and due to its novelty, only a few studies with limited numbers, mainly focusing on the elderly population have investigated its safety and outcome profile.^20-25^ Studies on non-osteoporotic fractures are lacking and we hypothesize that the SpineJack®-system is safe and efficient in the treatment of both, osteoporotic and non-osteoporotic vertebral fractures.

Thus, the aim of this single-center retrospective cohort study was to compare the usage, safety and efficacy of the SpineJack®-system as well clinical outcomes and peri- and postoperative complications between patients with tVCFs and oVCFs.

## Material and Methods

### Patients

All patients with vertebral fractures who were treated with percutaneous stent-kyphoplasty (SpineJack®) at the University Hospital of Zurich from November 2014 to December 2022 were assessed. All vertebras were operated with bilateral implants (two SpineJack® implants per vertebra).

Inclusion criteria were (a) treatment with percutaneous stent-kyphoplasty (SpineJack®) for a tVCF or oVCF; (b) signed general consent.

Exclusion criteria were (a) treatment with percutaneous stent-kyphoplasty (SpineJack®) for any other condition (e.g., neoplasm-related fracture); (b) missing general consent.

In total, 438 patients were operated with the SpineJack®-system during the assessed time frame of which 107 patients were excluded due to missing general consent and 21 patients were excluded due to neoplasm-related fractures resulting in a total of 310 assessed implants.

### Classification of Fractures

The assessed fractures were classified by two authors (JS, MK) independently using preoperative standing x-rays or CT-imaging in case no X-ray was available. In case of disagreement, consensus was reached. Osteoporotic fractures were defined as low-energy trauma and/or pre-existing diagnosis of osteoporosis in the patient`s chart. TVCFs were classified according to the AO Spine Thoracolumbar Injury Classification System,^7^ whereas oVCFs were classified according to the AO Spine-DGOU Osteoporotic Fracture Classification System.^10^

### Statistical Analysis

Statistical analysis was performed using SPSS 26® for Mac (SPSS, Chicago, Illinois, USA). Data is presented as frequencies (n) and means with the standard deviation (SD). To assess differences between groups, a Chi-Square test was used for nominal data. To assess differences in means between the two groups, an independent-samples *t* test was used for normally distributed continuous data. For not normally distributed continuous data, the Mann-Whitney-U test was used (Shapiro-Wilk ≤0.05). The non-parametric median test was utilized to evaluate differences between the groups analyzing ordinal data (e.g., visual analogue scale). A subgroup analysis was performed for stand-alone SpineJack®-procedures and procedures with SpineJack® and additional implant stratified by traumatic vertebral fractures (tVCFs) vs osteoporotic vertebral fractures (oVCFs). The level of statistical significance was set at *P* < 0.05.

### Ethical Considerations

This study was carried out in accordance with the local institutional ethics committee’s terms of reference (Kantonale Ethikkommission Zürich, Switzerland. BASEC-Nr. 2017-00408).

## Results

### Demographics

The majority of all 310 assessed implants were from male patients (n = 167, 53.9%). The mean age amongst all patients was 61.5 years (range from 16 to 93 years, SD 18.2) with a significant difference between male (59.3 years, SD 18.2) and females (64.0 years, SD 17.9) (*P* = 0.016, Shapiro-Wilk <0.001). All fractures involved either the thoracic (T1-T10) or lumbar (L3-L5) spine with most fractures affecting the thoraco-lumbar junction (T11-L2; 60.3%), followed by lumbar region (20.3%) and thoracic region (19.4%). Most of the assessed fractures were due to low-energy trauma (51.9%) with a significant difference between the sex (male 41.3%, female 64.3%) (*P* = <0.001). There was no difference between the trauma mechanism and the involved spinal region (*P* = 0.487). The minority of patients (32.9%) underwent additional dorsal instrumentation, with a significant higher rate of additional dorsal instrumentation in patients with high-energy trauma (55.0%) compared to patients with low-energy trauma (12.4%) (*P* = <0.001). There was no significant difference in the appliance of a dorsal instrumentation between the injured regions of the spine (*P* = 0.535). The majority of the fractures were oVCFs (50.6%), followed by tVCFs (49.4%).

### Characteristics of Osteoporotic and None-Osteoporotic (Traumatic) Fractures

The distribution of osteoporotic and non-osteoporotic fractures stratified by the AO Spine-DGOU OF classification and the AO Spine classification^7,8^ are depicted in Table 1.

**Table 1.** Distribution of Osteoporotic and Non-Osteoporotic Fractures Stratified by the AO Spine-DGOU of Classification and the AO Spine Classification.

n, %	Of Classification	n, %	AO Spine Classification
16, 10.2%	Of 1	38, 24.8%	A1
45, 28.7%	Of 2	10, 6.5%	A2
37, 23.6%	Of 3	63, 41.2%	A3
55, 35.0%	Of 4	42, 27.5%	A4
4, 2.5%	Of 5	-	-
157		153	

The mean age between the two groups differed significantly (mean age non-osteoporotic fractures: 49.6 years, SD 16.4 vs mean age osteoporotic fractures: 73.3 years, SD 10.5, *P* < 0.001, Shapiro-Wilk <0.001).

There was neither a significant difference in the distributional pattern between male and females for the AO Spine classification (*P* = 0.209) nor for the OF-classification. (*P* = 0.253) Significantly more high-energy trauma cases occurred in the non-osteoporotic group (96.7%) vs 0.6% in the osteoporotic group (*P* < 0.01).

There was no significant difference in the affected spinal level between the groups (43.8% thoracic level for none-osteoporotic fractures and 43.3% for osteoporotic fractures, *P* = 0.932).

### Surgery

The distribution of procedures in the assessed fractures stratified by sex is depicted in Table 2. The distribution of procedures in the assessed fractures stratified by fracture classification (osteoporosis vs non-osteoporosis) is depicted in Table 3.

**Table 2.** Distribution of Procedures in the Assessed Fractures Stratified by Sex.

Procedure	n Female	n Male	n Total	*P*-value
Stand-alone SpineJack®	73	61	134	**0.006**
SpineJack® + instrumentation	33	42	75	0.794
SpineJack® + other Vertebro-/Kyphoplasty	34	58	92	0.058
SpineJack® + other Vertebro-/Kyphoplasty + instrumentation	10	20	30	0.167

Bold = statistical significance (*p < *0.05).

**Table 3.** Distribution of Procedures in the Assessed Fractures Stratified by Fracture Classification (Osteoporosis Versus None-Osteoporosis).

Procedure	OF	AO Spine	n total	*P*-value
Stand-alone SpineJack®	77	51	128	0.05
SpineJack® + instrumentation	19	55	74	**<0.001**
SpineJack® + other Vertebro-/Kyphoplasty	59	21	80	**<0.001**
SpineJack® + other Vertebro-/Kyphoplasty + instrumentation	2	26	28	**<0.001**

Bold = statistical significance (*p < *0.05).

The mean surgery durations stratified by intervention are depicted in Table 4.

**Table 4.** Mean Surgery Durations Stratified by Intervention.

Procedure	Mean (min)	SD	Range (min)
Stand-alone SpineJack®	33.76	22.77	15-184
SpineJack® + instrumentation	131.40	72.59	56-428
SpineJack® + other Vertebro-/ Kyphoplasty	59.13	22.83	30-120
SpineJack® + other Vertebro-/ Kyphoplasty + instrumentation	149.07	56.22	80-315

### Clinical Parameters

**Figure 1.**
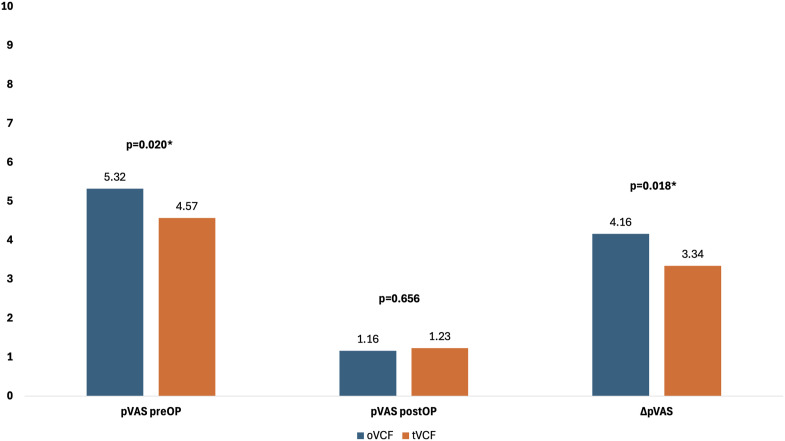
Depiction of mean pVAS preoperatively , postoperatively and mean reduction of pVAS of oVCFs vs tVCFs.

There was existing data of the pVAS (pain visual analogue scale) at admission and postoperatively (discharge day) in 271 fractures. The mean overall pVAS at admission of the whole collective was 4.9 (SD 2.7) vs 1.2 (SD 1.2) postoperatively. The mean overall pVAS reduction from admission to postoperative was 3.8 (range −3 to 10, SD 2.7). Figure 1 shows the mean pVAS preoperatively, postoperatively and mean reduction of pVAS of oVCFs vs tVCFs. Table 5 shows the mean pVAS preoperatively, postoperatively and mean reduction of pVAS of oVCFs vs tVCFs stratified by procedure. Patients with oVCFs who underwent SpineJack® and dorsal instrumentation showed a significantly higher preoperative pVAS compared to patients with tVCFs. (*P* = 0.033)

**Table 5.** Comparison of Mean Preoperative and Postoperative pVAS and Mean Reduction of pVAS Between oVCFs and tVCFs Stratified by Procedure.

Procedure	pVAS^preOP^			pVAS^postOP^			pVAS^red^		
	oVCF	tVCF	*P*-value	oVCF	tVCF	*P*-value	oVCF	tVCF	*P*-value
Stand-alone SpineJack®	4.9 (2.5)	4.2 (2.9)	0.211^a^	1.3 (1.2)	1.1 (1.1)	0.426^a^	3.5 (2.4)	3.2 (2.9)	0.355^a^
SpineJack® + instrumentation	5.9 (2.1)	4.4 (2.9)	**0.033** ^a^	1.2 (1.1)	1.3 (1.5)	0.906^a^	4.7 (2.3)	3.2 (3.0)	0.061
SpineJack® + other Vertebro-/Kyphoplasty	5.7 (2.6)	4.7 (3.1)	0.159^a^	1.0 (1.3)	1.1 (1.3)	0.525^a^	4.7 (2.7)	4.2 (2.3)	0.484^a^
SpineJack® + other Vertebro-/ Kyphoplasty + instrumentation	6.3 (1.8)	4.8 (1.9)	0.259^a^	1.0 (1.4)	1.2 (1.1)	0.735^a^	5.3 (3.2)	3.4 (1.9)	0.232

^a^Shapiro-Wilk<0.05 (Mann-Whitney-U test); pVAS^preOP^: mean preoperative pVAS; pVAS^postOP^: mean postoperative pVAS; pVAS^red^: mean reduction of pVAS. Bold = statistical significance (*p < *0.05).

The OF subtypes did not show any significant difference of the pVAS at admission (*P* = 0.178, median 5.00) as well as postoperatively (*P* = 0.586, median 1.00).

There was also no significant difference between the AO subtypes in terms of pVAS at admission stratified by fracture type (*P* = 0.426), median 5.00) as well as postoperatively (*P* = 0.190, median 1.00).

There was no statistical difference in mean pVAS reduction between stand-alone SpineJack®-procedures (3.41, SD 2.61) and procedures with additional implants (4.14, SD 2.78).

**Figure 2.**
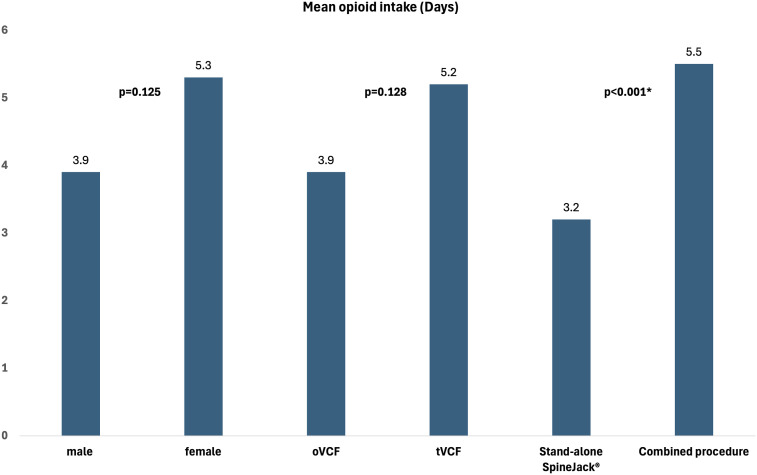
Depiction of mean length of opioid intake stratified by sex, fracture type and type of procedure.

The length of opioid intake was recorded in 327 fractures and averaged 4.6 days (range 0 to 72 days, SD 7.2 days). Figure 2 shows the mean length of opioid intake stratified by sex, fracture type and type of procedure.

**Figure 3.**
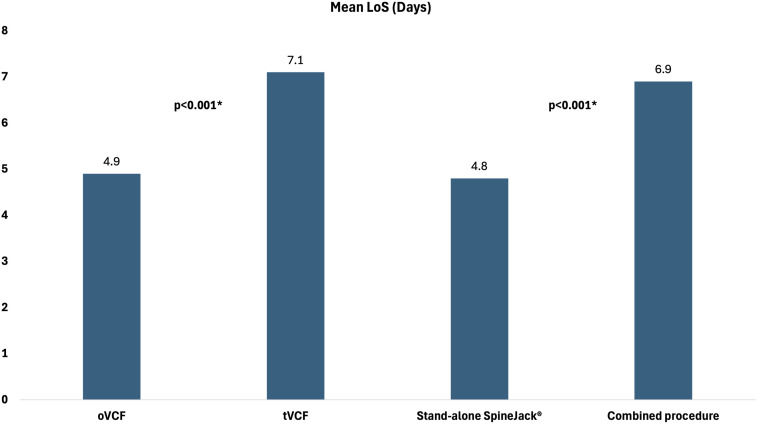
Depiction of mean LoS stratified by fracture type and procedure type.

The length of stay (LoS) was recorded in 328 fractures and averaged 6.1 days (range 0 to 72 days, SD 7.2). Figure 3 shows the mean LoS stratified by fracture type and procedure type.

### Complications

Of the whole collective, two fractures had intraoperative complications of which one occurred in the stand-alone SpineJack® group, and one occurred in the SpineJack®-Kyphoplasty-Instrumentation group.

There was one technical (SpineJack® implant-related) issue in the SpineJack®-Kyphoplasty or Vertebroplasty group, resulting in a dorsal dislocation of the implant during the retraction of the instruments.

In 29 of the SpineJack® implants there was intraoperative cement leakage detected (8.8%). Amongst the whole collective, no conversion of the SpineJack® implant was assessed (e.g., to ballon-kyphoplasty or dorsal instrumentation).

Postoperative neurological symptoms were seen in two cases (1.5%) after stand-alone SpineJack® kyphoplasty, while no neurological symptoms were observed in the SpineJack® + Instrumentation group.

A surgical site infection occurred postoperatively in four fractures (1.2%), which were seen in the SpineJack® with additional instrumentation group (50%) and by SpineJack® with additional instrumentation and kyphoplasty or vertebroplasty (50%).

## Discussion

The SpineJack® System is approved for the treatment of vertebral compression fractures Type A according to the AO Spine classification, as well as for pathological fractures and malignant lesions. The System is designed to provide robust support and rapid pain relief. The aim of this study was to compare the usage, safety and efficacy of the SpineJack®-system as well as clinical outcomes and peri- and postoperative complications between patients with tVCFs and oVCFs. Our findings suggest that SpineJack® kyphoplasty is not only effective for traumatic vertebral compression fractures and osteoporotic vertebral fractures but also holds significant potential as an adjunctive procedure in more complex spinal injuries.

SpineJack® kyphoplasty is considered a relatively fast procedure with an average operating time of 33.7 minutes. In a study by Vanni et al the average operating time was reported to be 40 minutes for SpineJack® kyphoplasty and 45 minutes for Ballon-Kyphoplasty. Similar times for Balloon-Kyphoplasty have been reported in studies by Dohm et al and Garnier et al. In contrast, vertebroplasty is generally associated with shorter operating times on average.^16,26^ Noriega et al investigated a mean operating time of 23 minutes for SpineJack® kyphoplasty and 32 minutes for ballon kyphoplasty.^17^ This demonstrated the efficiency of the SpineJack® procedure in clinical practice.

In our cohort, a significant reduction in pain was observed postoperatively.

From admission to the day of discharge, patients experienced a mean overall pain reduction of 75.75%, as indicated by a decrease in pVAS scores from 4.9 upon admission to 1.2 postoperatively. Notably, patients with osteoporotic fractures demonstrated a greater reduction in pain, with a 78% decrease in pVAS scores (from 5.32 at admission to 1.16 postoperatively), compared to those with non-osteoporotic fractures, who exhibited a mean pain reduction of 73% (pVAS admission 4.57 vs 1.23 postoperatively).

Our study demonstrates that these data are highly comparable to the findings of Noriega et al, who reported a postoperative pain reduction of 72% in their cohort as well as to the study by C. Renaud which observed a pain reduction of 77% in patients with traumatic or osteoporotic vertebral compression fractures.^27,28^

A meta-analysis from 2006 by Hulme et al, demonstrated that vertebroplasty achieved an average pain reduction of 63.4% and kyphoplasty 52.5%. Unfortunately, they did not assess the timing of last the pVAS assessment for these calculations. Taking this into account and consider our immediately postoperative measurement, it can be assumed that the pain reduction achieved through SpineJack® kyphoplasty is at least not inferior compared to balloon-kyphoplasty or vertebroplasty as shown in several studies and in addition, might be even higher.^29,30^

Furthermore, our study demonstrated that patients undergoing stand-alone SpineJack® procedures require significantly shorter durations of opioid analgesics postoperatively comparted to those treated with combined procedures, underscoring the procedures superior efficacy in pain management and its potential to reduce opioid dependency.

The mean hospital length of stay across all patients was 6.1 days. Patient with osteoporotic fractures had an average stay of 4.9 days, whereas those with traumatic fractures stayed for an average of 7.1 days. Notably, patients who underwent stand-alone SpineJack® procedures (mostly the osteoporotic group) exhibited a significantly shorter hospital stay averaging 4.8 days compared to those who received combined surgical interventions. Similarly, C. Renaud reported a mean hospital stay of 3.7 days following SpineJack® treatment in his study.^28^ Consistent with the literature, longer hospitalization durations were observed for traumatic fractures.^31,32^

In terms of complications, the SpineJack® kyphoplasty is a safe procedure. Immediate postoperative assessments indicated no major procedure-related complications. Systemic adverse events and postoperative neurological deficits were not linked to the SpineJack® implant but rather to general poor health and the fracture pattern. Also, the observed surgical site infections were likely due to the additional procedures rather than the SpineJack® implant itself. There was only one implant-related complication (0.3%) resulting in a dorsal dislocation of the SpineJack® implant during the retraction of the instruments. Renaud also reported one technical implant-related issue in his study.^28^

Cement leakage is the most observed complications of the intervention (8.8%). All these cement extrusions remained without clinical relevance. Noriega et al reported a similar cement leakage rate of approximately 7.8%, and the majority were asymptomatic.^17^ Premat et al investigated intradiscal leaks without clinical consequence in 36.8% of the patients.^33^ Renaud reported cement leakage in 12 of 77 (15.6%) patients, with one case resulting in nerve-root damage.^28^ Numerous studies report cement leakage rates for ballon-kyphoplasty to be less than 10%. In comparison, vertebroplasty is generally associated with higher leakage rates, often exceeding 40%.^29,34,35^ In summary, it can be stated that cement leakages are in the short term without clinical consequence, and SpineJack® kyphoplasty demonstrates similar outcomes to ballon kyphoplasty regarding the incidence of cement extravasation.

### Limitations

This study has several limitations that must be acknowledged. One of the primary limitations of this study is its retrospective design, which inherently carries a risk of selection bias. The study focuses on perioperative and immediate postoperative outcome, without addressing long-term results. Additionally, the inclusion of patients who underwent kyphoplasty in combination with other interventions complicates the interpretation of results. The lack of long-term follow-up data further limits our understanding of potential late complications and the sustained effectiveness of the intervention. While radiological parameters such as postoperative height as well as kyphosis restoration were not assessed in this study, we aim to perform an assessment of these parameters in a separate analysis.

Further, a prospective study addressing the above-mentioned shortcomings will be performed to gain further insights.

## Conclusion

Percutaneous stent-kyphoplasty (SpineJack®-system) appears to be a safe and effective therapeutic option, whether used as a stand-alone procedure or in combination with other interventions, for both osteoporotic and non-osteoporotic fractures, providing a relatively fast pain relief and shows a low complication rate.

Future prospective studies with longer follow-up periods and more controlled patient population are needed to provide a more comprehensive assessment.

## Data Availability

Data is available upon reasonable request towards the corresponding author.*
